# Supplementary material for: Sorting at embryonic boundaries requires high heterotypic interfacial tension
Source: Nat Commun. 2017 Jul 31;8:157. doi: 10.1038/s41467-017-00146-x (PMC5537356; doi:10.1038/s41467-017-00146-x)
Supplement: Supplementary file 2 — Supplementary Software 1 [file 41467_2017_146_MOESM2_ESM.zip › PottsModel/SrcPottsModel/doc/index-files/index-23.html]

W-Index


---


|  |  |  |
| --- | --- | --- |
| **Overview** \| Package \| Class \| Use \| **Tree** \| **Deprecated** \| **Index** \| **Help** | |  |
| **PREV LETTER**   **NEXT LETTER** | **FRAMES**    **NO FRAMES**     **All Classes** |  |


A B C D E F G H I J K L M N O P Q R S T U V W X Y Z 

---


## **W**

**width** - Variable in class utils.HSLColor.ColorScaleCanvas: **WITHIN\_CELL** - Variable in class model.Constants.ParameterManager: **withinBounds(double, double)** - Method in class gui.PottsCanvas: **withinBounds(Coordinates)** - Method in class gui.PottsCanvas: **withinBounds(int, int)** - Method in class model.Lattice: **wrapUp(int)** - Method in class engine.EnergyStatistic: **wrapUp(int)** - Method in class engine.Statistic: Call at the end of a MCS **wrapUp()** - Method in class engine.StatisticsManager: Make sense out of the observations we collected during the MCS. **writeLattice(Lattice, String)** - Static method in class model.LatticeUtilities: **writer** - Variable in class engine.StatisticsManager: **writeToFile** - Variable in class engine.StatisticsManager

---


|  |  |  |
| --- | --- | --- |
| **Overview** \| Package \| Class \| Use \| **Tree** \| **Deprecated** \| **Index** \| **Help** | |  |
| **PREV LETTER**   **NEXT LETTER** | **FRAMES**    **NO FRAMES**     **All Classes** |  |


A B C D E F G H I J K L M N O P Q R S T U V W X Y Z 

---
